# Flood Hazard Zoning of Upper Awash River Basin, Ethiopia, Using the Analytical Hierarchy Process (AHP) as Compared to Sensitivity Analysis

**DOI:** 10.1155/2023/1675634

**Published:** 2023-04-10

**Authors:** Tewodros Mulu Mekonnen, Addisalem Bitew Mitiku, Abel Tadesse Woldemichael

**Affiliations:** Ethiopian Construction Design and Supervision Works Corporation, Addis Ababa, Ethiopia

## Abstract

Floods and droughts have been two of the most devastating consequences of the climate crisis affecting billions of people in the world. However, unlike the other natural hazards, flooding is manageable through appropriate flood management mechanisms. This study emphasizes on developing a flood hazard zone for the Upper Awash River Basin (UARB), Ethiopia. Six relevant climate, physiographic, and biophysical factors were considered. Then, a flood hazard map was developed employing the analytic hierarchy process (AHP) method and further validated using sensitivity analysis and collected flood marks. The results revealed that drainage density, rainfall, and elevation have higher significance, while land use and soil permeability have a low impact in the process of flood generation. The map showed vulnerable areas at different levels and can serve as a valuable input for the decision makers to consider in the process of implementing emergency plans as well as long-term flood mitigation options.

## 1. Introduction

A warmer climate, with its increased climate variability, will increase the risk of both floods and droughts [1–6]. The effect of a warmer climate is more pronounced by urbanization and industrialization. The urbanization usually leads to unprecedented deforestation and land use and land type changes. According to Stoy [7], deforestation often increases land-surface and near-surface temperatures and the severity of extreme heat. These results in a decline in land perviousness and increase in the amount of sunlight reflected back from the earth's surface. As a result, the atmosphere warms up; a lot of water evaporates from seas, oceans, as well as from any water resources located on the earth, which in turn creates a feedback loop between global climate change and extreme hydrological events such as flooding and drought [8–10].

Floods cause serious harm to people and adversely affect socioeconomic development around the world, especially in urban areas where the high risks of flooding are the consequences of urbanization and industrialization [11–15]. The World Bank report [16] states that over the last two decades, floods and droughts—two of the most devastating consequences of the climate crisis—have affected 3 billion people, with staggering costs in human suffering and economic loss. Another report of the United Nations (UN) reveals that floods alone affected 2.3 billion people from 1995 to 2015 in the world [17]. This contributes to 56% of the total affected people by weathered-related disasters. The report also states that there were 3062 flood disasters in those mentioned years that accounted for 47% of all geophysical hazards occurred in the globe. Rentschler et al.[4] also revealed that 1.81 billion people, which share 23% of the world population, are directly exposed to 1-in-100-year floods. Flood events are becoming more severe, and flood frequency has been rising causing mainly a distraction of agricultural areas and food supply sectors and exacerbating malnutrition problems of the poorer areas of the world, such as Asia and Africa [14].

In Ethiopia, flash flooding, which is mostly anticipated in the areas of a river side as well as in areas with low water percolation capacity, has been pronounced in the southern, north-eastern, and eastern parts of the country [18]. Flood damaged and displaced hundreds thousands of people in Ethiopia (https://floodlist.com/tag/ethiopia). According to EM-DAT [19], floods and droughts were severe national disasters of the country during the last centuries causing huge loss of human lives and properties. Historical records on flood data suggest that Ethiopia faced 47 major floods since 1900, which affected close to 2.2 million people [20]. Mamo et al. [21] reported that the frequency of flood occurrences in the country increased from decade to decade with the 2001–10 decade being the most flooding decade with five flood years out of ten, whereas the last 2011–20 decade witnessed three flood years.

The Awash River Basin (ARB) with a total land area of 110,000 km^2^ is one of the major river basins in Ethiopia that has series flooding problems [22–25]. An approximate area in the order of 200,000–250,000 ha is subjected to flooding during high flows of the Awash River. The Upper Awash River Basin (UARB), which is the subject of this study, constitutes part of the ARB and is subjected to intense flooding for short durations after strong or prolonged rainfall events. Recurrent flooding that has occurred in UARB has been a critical problem. The flood event usually occurs in summer (“kiremt”) season of the country, Ethiopia. According to a socioeconomic study conducted in the basin, in 2017/2018, floods affected 8,477 households, more than 18,996 ha of agricultural land, 25,087 live stokes, infrastructures, and health and educational institutions. Schools in the UARB often started late because the flooding and health centers are not functional in the rainy season of the country.

Unlike most types of disasters such as volcanoes and earthquakes, floods are preventable and manageable through proper implementation of an integrated flood management approach and proper mitigation measures. Flood hazard zoning provides a starting point and useful resource for flood risk management, mitigation actions, and governance. Moreover, flood hazard zone maps are a valuable tool in planning the future development of the city, as well as identify areas that need development of infrastructure and flood drainage [26].

The main purpose of this study is to map flood hazard zones in UARB by implementing the analytical hierarchy process (AHP) method. The AHP method is a multicriteria analysis approach for organizing and analyzing complex decisions based on mathematics and psychology. It was developed by Saaty in the 1970s and has been extensively studied and refined ever since [27, 28]. The AHP approach for flood hazard mapping is gaining wide range recognition in recent times. Hadjimitsis et al. [29] implemented the AHP to compare the different factors and their relative importance in assessing natural and anthropogenic risk of culture heritage in Cyprus. Fernandez and Lutz [30] zoned Bwana Argentina Yerba city in terms of flood risk using GIS and a multicriteria decision-making system (AHP). Kazakis et al. [31] used the AHP approach to assess flood hazard areas on a regional scale in north-eastern Greece, where recurring flood events have appeared. Similar other studies can be found in the works of [32–36].

Besides the AHP process employed in this study, the research applied the sensitivity analysis method (i.e., map removal sensitivity analysis techniques as it was discussed in [35, 36]), and results were validated against the collected flood marks. In both AHP and sensitivity methods, six relevant climate, physiographic, and biophysical factors that were essential for flood hazard zoning were identified and used. These factors include rainfall amount, slope, elevation, river density, land use, and soil type-based permeability. Then, these factors were used to rank the level of importance of each of them in the process of flood generation. The study is organized as follows: Section 2 presents the study region.  Section 3 presents the data and methodology used in this study.  Section 4 discusses the findings. Finally, Section 5 gives the conclusion and recommendations of the work.

## 2. Study Area

**Figure 1:**
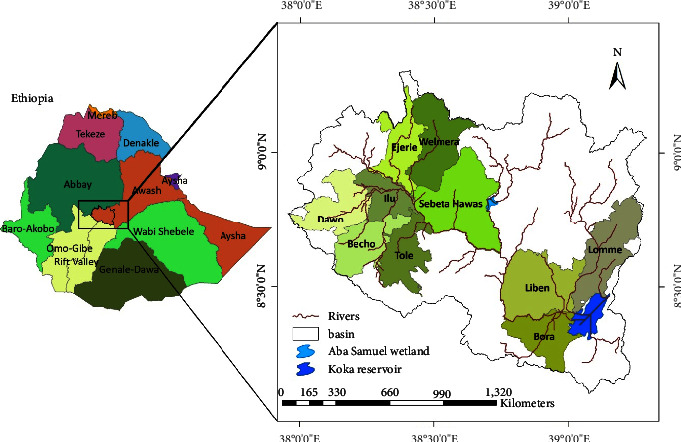
Location map and flood affected districts of the UARB.

The Upper Awash River Basin (UARB) is the upper part of the Awash River Basin (i.e., one of the 12 major basins) of Ethiopia covering about 11,607 km^2^. The basin is surrounded in the north by the Abbay basin, in the west and southwest by Omo Gibe and Rift valley lake basins, and in the east-south by the Wabi Shebele basin. The river originates from the south of Mount Warqe at an altitude of 3000 m above mean sea level (m.s.l.) at a place specifically called Elam close to the Ginchi town and runs up to Koka reservoir with altitude about 1500 m above m.s.l. The Upper Awash River has meandering characteristics traveling about 200 km until it reaches to Koka reservoir (Figure 1).

The major tributaries of the river include Holeta, Alito, Teji, Gilo and Kelina, Kebena, Great and Little Akaki, and Mojo rivers. The basin consists of eight flood affected districts such as Ilu, Dawo, Sebeta Hawas, Welmera, Ejere Ejersa Lefo, Liben-Chuquala, and Bora districts (Figure 1). It has a bimodal rainy season. The main rainy season lasts from June to September and the second minor rain occurs from March to April. The basin receives an average annual rainfall of 1052 mm where it varies from 400 mm to 1900 mm per year from place to place. The mean annual temperature ranges from 20.8°C to 29°C at the Koka dam [25].

Recent history has shown that the basin is highly affected by recurrent floods as well as erosion [22, 39–42]. Increasing agricultural activities without land conservation and overgrazing leads to erosion and further aggravates flooding. The cumulative effect of these hazards warrants for the need of proper planning for disaster reduction and sustainable mitigation plans.

## 3. Data and Methodology

In this study, six parameters such as rainfall, slope, elevation, river density, land use, and soil type-based permeability were considered to map the flood hazard zones. Space Shuttle Radar Topography Mission (STRM) data were used to determine terrain elevation, and consequently slope and drainage density were extracted from the digital elevation model. The land use and land cover (LULC) data of the study area were obtained from the Ethiopia Geospatial Mapping Agency (EGMA) for the year 2013. It showed that a large portion of the basin is covered by agricultural land such as annual and perennial cropland sharing 63.1% and 8.9%, respectively, while shrub land and grassland land cover 12.2% and 0.8% accordingly. The land use type also includes dense forest (i.e., dense, moderate, and spares type), woodland, settlements, bare soil, and water bodies. The soil map of the basin was collected from the Food and Agriculture Organization (FAO) soil database. The dominant soil types of the study area consist of Pellic Vertisols (46.2%) and Vertic Cambisols (12.8%). Luvic Phaeozems, Eutric nitisols, and Orthic Solonchaks also shares 7.3%, 6.3%, and 6.2%, respectively. The spatial variability of the collected data was prepared in raster format and further classified accordingly. Elevation, drainage density, slope, and rainfall were classified in accordance to get a uniform interval in between classes (Table 1). Similar research studies also applied uniform intervals to categorize classes for different factors [41–44]. The land use type of the basin was condensed into five classes such as forest, woodland, grass land, cultivated land, and water body where their level of influences for flood occurrence varies from very low, low, moderate, and high to very high, respectively. Similarly, according to the Soil Conservation Service (SCS) classification, the soil type was categorized into four hydrological soil groups (A, B, C, D) [45, 46] and water bodies.

### 3.1. Analytical Hierarchy Process (AHP)

Flood hazard zoning is developed for UARB using the analytical hierarchy process (AHP) and further elaborated using the applying sensitivity analysis method. The AHP method uses a multicriteria analysis approach, which was developed by Saaty in the 1970s [27, 28], and has been extensively used ever since [41–44, 47–52]. This allows for selecting potential parameters that cause flooding. When quantitative ratings are not available or difficult to rate factors, decision makers can still recognize whether one criterion is more important than another using pairwise comparison similar to what Saaty [28] developed. In this study, weight for each factor was also determined using a pairwise comparison matrix.

The AHP includes a comparison of importance between factors, normalization, and computing consistency ratio. The relative importance of one factor over the others was defined based on the rating scale provided in Saaty [28], as given in Table 2. The relative significance between the criteria is evaluated from 1 to 9, where 1 indicates equal important criteria and 9 represents much more important criteria.

The pairwise comparative weight is highly dependent on expert judgment and should fulfill the consistency ratio (CR) criteria. The normalization of weights assigned to each parameter is done using Eigen vector and further validated for consistency check employing CR formula (equation (1)). The value obtained from the CR must be less than or equal to 0.1 [53]; as a result, any subjectivity which might be involved during prioritizing the level of importance of one factor over the other can be reduced.

(1) $$\begin{array}{c} CR=\frac{CI}{RI}\text{,} \end{array}$$

where CI represents the consistency index (**CI**=average number of consistency vector − **n**/**n** − 1) and computed from the pairwise comparison matrix of all the parameters. Whereas, RI is the random consistency index as stated in Saaty [53], where value depends on the number of factors (*n*).

### 3.2. Sensitivity Analysis Method

The sensitivity analysis was also performed considering six flood-generating factors such as rainfall, slope, elevation, river density, land use and soil type-based permeability, and weight overlay spatial analysis techniques. It is a biased-free map removal analysis technique. Initially, all factors are considered as if they are equally important for generating floods. As a result, a base-case scenario was formulated providing equal weight for each factor (i.e., 16.67% weight for each). And then, an additional six scenarios were developed by turning off/removing one factor at a time. Consequently, a correspondent flood hazard map was produced for each scenario. Thereafter, the flood hazard maps of different scenarios were compared with the base-case scenario and the weights of each factor were determined accordingly.

**Figure 2:**
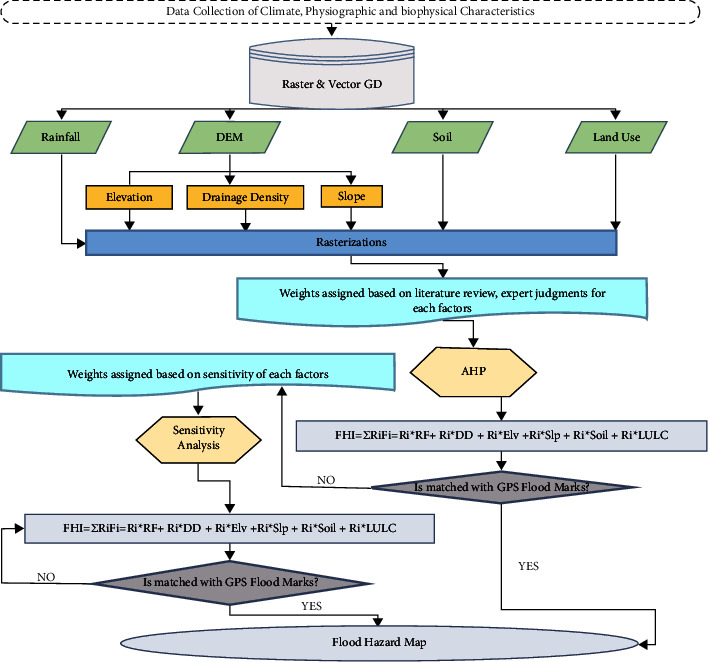
Flowchart for the flood hazard zoning procedure.

The flood-generating factors with classified raster layers were then multiplied with correspondent weights that were obtained from the AHP method and later validated by the weights derived from the sensitivity method. Finally, these layers were overlain to generate flood hazard zones following equation (2) and the steps involved in Figure 2.

(2) $$\begin{array}{c} FHI=\overset{n}{\underset{i=1}{\sum }}R_{i}F_{i}=R_{i}RainFall+R_{i}Drainage\;Density+R_{i}Elevation+R_{i}Slope+R_{i}Soil+R_{i}LULC\text{,} \end{array}$$

where FHI represents the flood hazard index, R_i_ are the corresponding weights, and F_i_ are the flood-generating factors.

## 4. Results and Discussion

### 4.1. Classifications of Flood-Generating Layers

**Figure 3:**
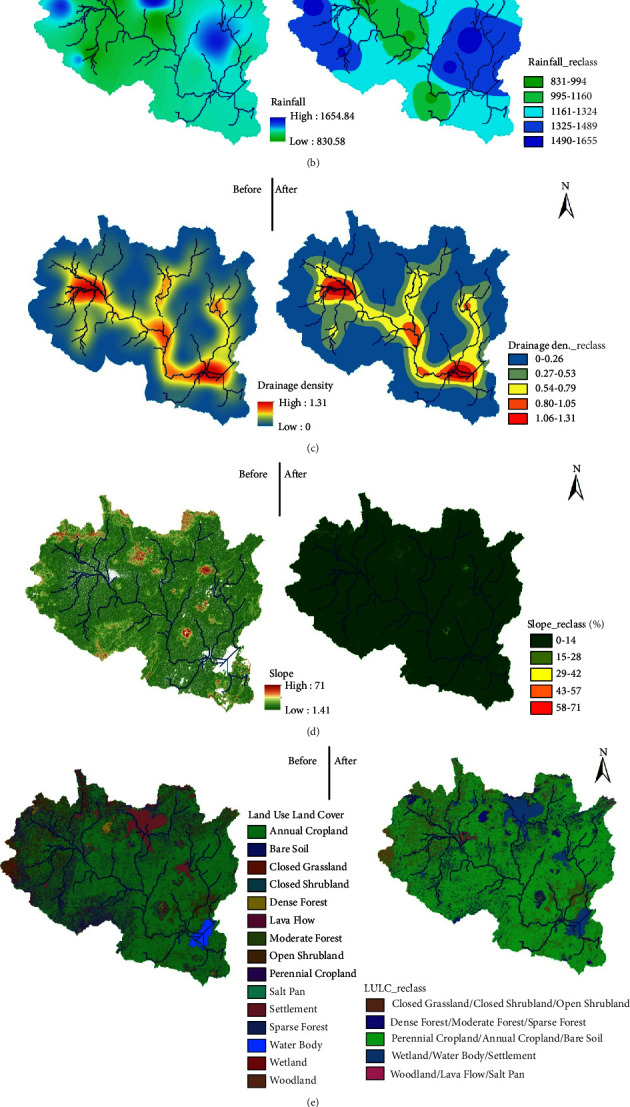
Classification of flood-generating layers. (a) Elevation layer. (b) Rainfall layer. (c) Drainage density layer. (d) Slope layer. (e) Land use land cover layer. (f) Soil layer.

Raster layers were prepared for each of the six flood-generating factors at the start of the process. These raster layers were further classified into five classes based on their flood-generating capability of the area and using an equal interval method of spatial analysis tool. Thus, a very high class was assigned a rate of 5, a high class rated as 4, a moderate class rated as 3, and low and very low classes rated as 2 and 1, respectively (Table 1 and Figure 3(a)).

Considering elevation as one of the flood-generating factors, the lowest elevation values indicate the highest possibility of flood-generating capability and hence are assigned the highest rate of five. From Table 1, this value ranges from 1580 m to 1978 m. The rest of the elevations between 1978 m and 3568 m were also categorized from high to very low depending on flood-generating potential (Table 1).

It is obvious that the rainfall amount has a direct relation with the amount of flood generated. In UARB, as the long-year mean rainfall pattern indicates, there is high precipitation in the east highlands and northwest and southwest peripheries, while there is low rainfall in the west lowlands and central part of the river basin (Figure 3(b)). As a result, those areas with the highest rainfall intensity ranging from 1490 mm to 1655 mm are given the highest rate of five and rated as very highly vulnerable areas (Table 1). Areas with rainfall amounts ranging from 831 mm to 1490 mm are accordingly classified as very low to high depending on the rainfall amount generated (Figure 3(b)).

The drainage density is the total length of all the streams and rivers in a drainage basin divided by the total area of the drainage basin. It was computed from the digital elevation model (DEM) of the basin applying Kernel density, and it varies from 0 to 1.31 for the basin (Table 1). Similarly, the drainage density layer was further classified in into five classes; a higher drainage density indicates a very high hazardous area and assigned a rate of five, whereas an area having a smaller drainage density results in the minimum area to be affected by flood and is ranked as very low (Figure 3(c)).

The slope is also computed from the DEM of the basin (Figure 3(d)). The general assumption followed was that the terrain with the steepest slope tends to retain the least amount of water, whereas terrains with the flattest slopes retain the more water. Accordingly, terrains with the flattest slope were identified as highly vulnerable to flood and assigned a rate of five (i.e., slope from 0 to 14%). Other slope values ranging from 14% to 71% are classified from high to very low rates.

The LULC of a basin plays a significant role in rain water movement either by retarding or accelerating overland flow. LULC highly influences the infiltration rate and thus the water partitioning between surface and groundwater systems of a catchment. Forest land enhances the infiltration capacity of the surface and as a result reduces flooding and hence is given the lowest rate of 1. Other land use types such as woodland, grassland, and cultivated land affect water percolation at different levels and are classified as low (rate 2), moderate (rate 3), and high (rate 4), respectively. Settlements and water bodies highly aggravate overland flow and thus flooding; as a result, they are assigned with the highest rate (rate 5) (Figure 3(e) and Table 1).

The soil type-permeability can amplify/extenuate the extent of flood events. Different soil types have different capacities to infiltrate water. Sandy soils have higher hydraulic conductivities than fine-textured soils because of the larger pore space between the soil particles. As such, the infiltration rate of clayey soils is much lower than that of sandy soils. As mentioned in the methodology section, the soil of the Upper Awash Basin was divided into four hydrologic soil groups based on infiltration rates (groups A, B, C, and D). Accordingly, the classification and rating of the soil factor were made as shown in Table 1.

### 4.2. Flood Hazard Map Using the AHP Method

The optimal pairwise diagonal matrix of the study is stated in Table 3. As mentioned in the methodology section, the pairwise diagonal matrix was developed by assigning the level of importance of one factor over the other. For instance, rainfall intensity is more important than land use and therefore assigned the value 7. The row describes the importance of land use (Table 3). Therefore, the row has the inverse value of the rainfall in the pairwise comparison (i.e., land use is 1/7^th^ as significant as the rainfall intensity). Various comparison matrices were assumed and their corresponding consistency ratios were computed until a satisfactory result, which is a CR of less than or equal to 0.1, was achieved.

Thereafter, as mentioned in the methodology section, percentages of preference values were computed by dividing the individual preference value of each factor over the cumulative preferences' values in a column (Table 4). Consequently, the ratio of preference values with weight (i.e., which are determined in percentage preference matrix) gave us the weight value matrix from which the average number of consistency vectors was determined as 6.477 and results in a consistency index of 0.0955. The random index (RI) depends on the size of the matrix, and when the matrix size is equal to 6, then RI will be 1.24 [53]. Following, by applying equation (1), CR is determined as 0.077 which is under the allowable threshold value.

**Figure 4:**
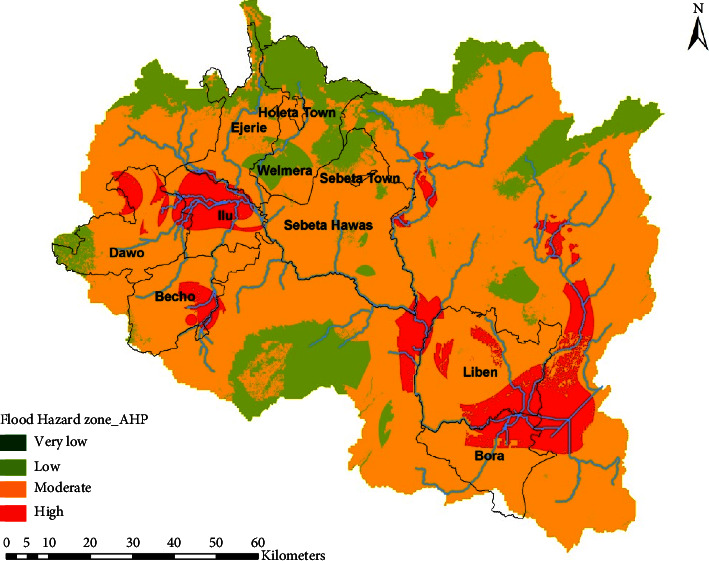
Flood hazard index map developed using AHP methods.

The weights of flood-generating factors were then computed as described in Table 5. The weight factors computed from AHP are applied for each of the flood-generating layers in equation (2), and the weighted layers were overlain one after the other to generate a flood hazard map of the basin (Figure 4). As shown in Figure 4, the high flooding zone matches with the sample flood marks collected in the years 2018 and 2019.

The flood hazard map (Figure 4) shows that the majority of the catchment has been subjected to a moderate flood hazard amounting for an estimated area of about 8352 km^2^ that accounts for about 76.42% of the total area of the UARB. The figure also shows that 1866 km^2^ of the basin area is vulnerable to a high flood hazard while 709 km^2^ (6.48%) was prone to a low flood hazard. Areas at the upstream of the basin (Ilu and Sebeta Hawas districts) as well as the final outlet of the river (upstream of Koka reservoir covering Bora and Liben districts) were more susceptible to high floods.

Excluding the “external” flood-generating factor which is rainfall, the AHP result reveals that drainage density and elevation have a high influence on flood occurrence. Similarly, Figure 4 shows that places with low elevation range and high drainage density are more vulnerable to high floods.

### 4.3. Flood Hazard Map Using the Sensitivity Method

**Figure 5:**
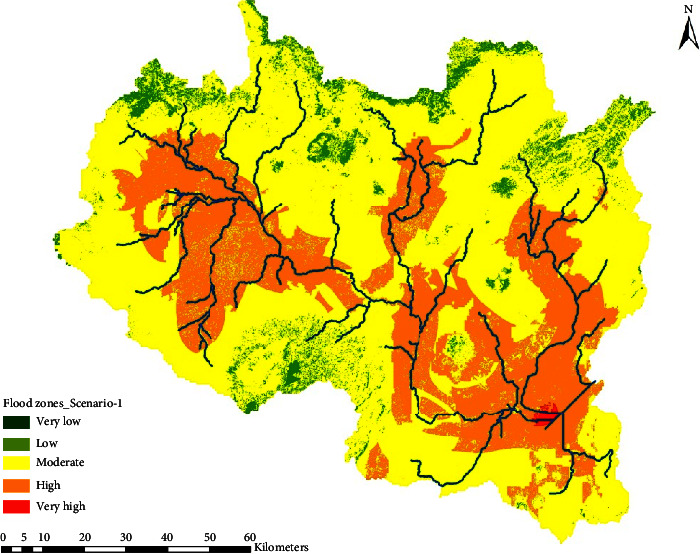
Flood hazard zone for the of base-case scenario (Scenario-1).

The flood hazard zone developed using the AHP method was further validated with sensitivity analysis. In the sensitivity analysis, all factors were initially considered as if they are equally important for generating floods and have given equal weight. And hence, the base-case scenario with a flood hazard map was developed given 16.67% weight for each of the flood-generating factors (Figure 5).

**Figure 6:**
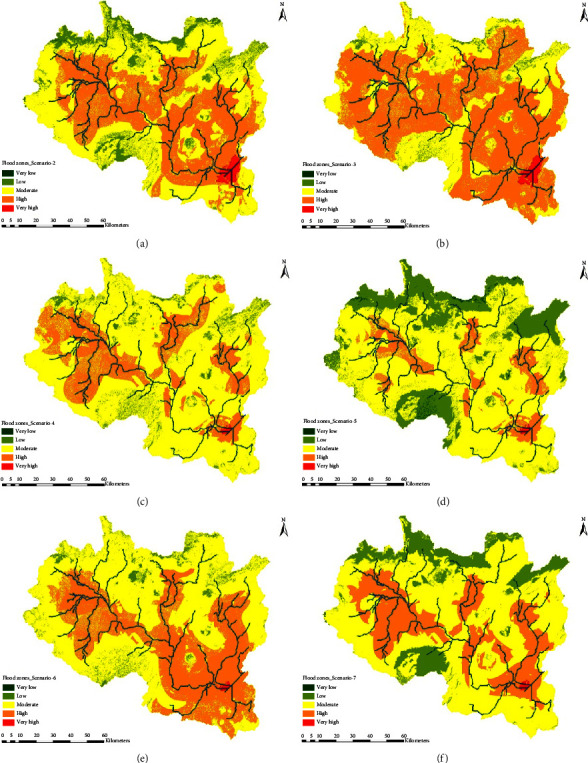
Flood hazard index maps of different scenarios.

Thereafter, additional six scenarios were performed by turning off one factor at a time (Table 6). When one factor is turned off, the remaining five factors will have a weight of 20% each. Then, the correspondent flood hazard maps were also determined for each scenario (Figure 6). An evaluation was performed for identifying the impact of each factor against the others. This helps in a better understanding of the importance of each factor in identifying against the low impact factors for flooding.

Each flood hazard map shown in Figure 6 exhibits the classification from very low to very high zones in spatial coverage. Consequently, the area coverages were computed from very low to very high ranges as given in Table 6. Examining the difference between flood hazard maps of Scenario-1 (Figure 5) and Scenario-2 (Figure 6(a)) helps us to determine the weight factor of rainfall.

Similarly, weights (significance) of other remaining flood-generating factors were computed referring to the base-case scenario and further validated against the GPS-tracked flood mark of 2018 and 2019 flood events.

**Figure 7:**
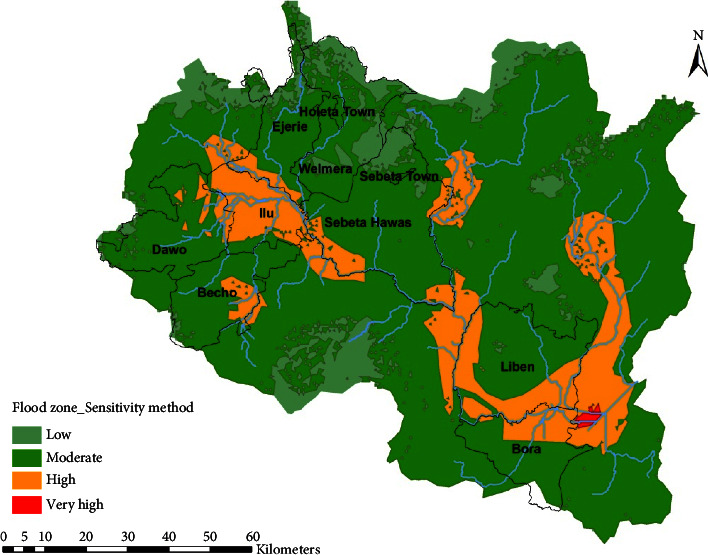
Flood hazard zone developed using sensitivity analysis.

The sensitivity analysis showed that a very high flood zone which leads to extreme events was observed due to drainage density (30%), rainfall intensity (25%), and elevations (15%), respectively, whereas LULC (10%) and soil types (5%) showed the lowest significance. Then, the newly computed weights were applied to the corresponding layers of flood-generating factors and resulted in a flood hazard map, as shown in Figure 7. The weighting values derived using sensitivity analysis were somehow similar resultant to those computed using the AHP method, showing rainfall, drainage density, and elevation have a higher influence for causing flood in both methods, respectively. Areas located in Ilu, Sebeta Hawasa, Bora, and Liben Chiquala districts are highly vulnerable to flood, while very high flood exposure exists in the upstream of Koka reservoir.

As mentioned, the sensitivity analysis is a biased-free analysis method and thus optimally enhances for computing reasonable weighting values. The techniques used in this analysis method are logical and by itself leads to the solution. The flood map derived using this method matches well with the flood hazard map derived by AHP. Thus, it can be used as a validating technique while developing flood hazard maps.

### 4.4. Validation Process

**Figure 8:**
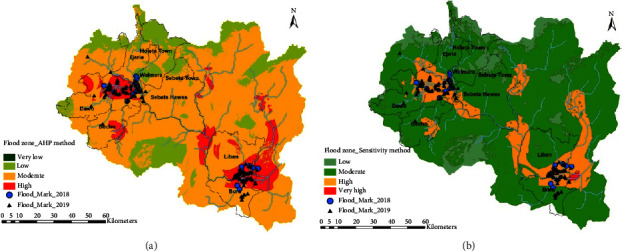
Overlaying collected flood marks over the developed flood maps.

The validation process was made by overlaying and comparing the traced peripheral of the flooded areas with the results achieved through the AHP method and sensitivity analysis method in the study area. A field visit has been made, and the peripheral of flood marks have been collected for the years 2018 and 2019 through Garmin hand GPS. A total of 174 flood marks were collected (i.e., 24 and 150 points for the year 2018 and 2019, respectively). The collection includes tracing the peripheral boundaries of flooded areas and taking their spatial location (Easting and Northing). Flood marks were collected for Bora, Liben Chiquala, Ilu, Dawo, Sebeta Hawasa, Ejere, and Ejere Lefo districts for the two successive years varying the spatial extent from the river channel. High flood extent was recorded in Ilu and Sebeta Hawas districts. Those boundaries of flood points then overlaid in the generated flood maps in ArcGIS environment (Figure 8). As shown in Figures 8(a) and 8(b), most of the flood marks laid over the high flood zone of the maps developed using AHP sensitivity analysis methods. From the total collected flood marks, 146 points (83.9%) laid over high flood zone of the AHP flood map, while 28 points laid over moderate flood zones. For the flood map developed using the sensitivity method, 158 flood marks, which is 90.8%, was laid over high flood zones, while 9.2% was spread over moderate flood zones. Overall, more than 83% of the collected flood marks match with high flood zones of the developed maps which revealed their reliability.

## 5. Conclusion

Recurrent floods have increased from year to year in UARBs and have been causing huge economic losses and associated social impacts. This is mainly due to climate and other associated changes. Urbanization, agriculture, and grazing land have increased in the basin which significantly reduces the permeability of the land and enhances overland flow. Consequently, this resulted in flooding in the basin year after year. Thus, developing accurate flood hazard zoning will favor the prevention and sustainable management of floods in the basin.

In this study, various flood-generating factors such as rainfall, slope, elevation, drainage density, land use, and soil type-based permeability were considered, and their corresponding influence was quantified using the AHP method and further validated by applying sensitivity analysis and previously collected flood marks in order to develop an appropriate flood hazard zone of the basin. Identification of the significant factor helps to select the type of measures to be employed while taking mitigation measures and thus enhances emergency flood adaptation mechanism and favors extreme flood management options.

The study reveals that drainage density, rainfall, and elevation have higher impacts on generating floods relative to the other factors. However, land use and soil permeability have a lower influence. Consequently, feasible flood hazard maps showing different flood zones such as high, moderate, low, and very low were developed and validated against flood marks and sensitivity analysis. The flood hazard map showed that places with lower elevation range and high drainage density were more susceptible to flooding. Specifically, places at Ilu, Sebeta Hawasa, Bora, and Liben Chiquala districts were more vulnerable to the recurrent flood. The results of the study can be instrumental for the decision-making parties for implementing emergency flood adaptation mechanisms as well as for implementing long-term sustainable extreme flood management options.

## Figures and Tables

**Table 1:** Class and rating of flood-generating factors.

Factor	Class	Flood vulnerability level naming	Rating
Elevation	1580–1977	Very high	5
	1978–2374	High	4
	2375–2772	Moderate	3
	2773–3170	Low	2
	3171–3568	Very low	1

Drainage density	0–0.26	Very low	1
	0.27–0.53	Low	2
	0.54–0.79	Moderate	3
	0.80–1.05	High	4
	1.06–1.31	Very high	5

Slope	0–14	Very high	5
	15–28	High	4
	29–42	Moderate	3
	43–57	Low	2
	58–71	Very low	1

Rain fall	831–994	Very low	1
	995–1160	Low	2
	1161–1324	Moderate	3
	1325–1489	High	4
	1490–1655	Very high	5

Land use	Forest	Very low	1
	Woodland	Low	2
	Closed grass land	Moderate	3
	Cultivated land	High	4
	Water body/settlement	Very high	5

Soil	A	Very low	1
	B	Low	2
	C	Moderate	3
	D	High	4
	Water body	Very high	5

**Table 2:** Fundamental scales of absolute numbers (“Saaty scale”), adapted from Saaty [53].

Level of importance	Descriptions
1	Equal importance of both factors
3	Judgment slightly favors one factor over another (moderate difference of importance)
5	Judgment strongly favors one factor over another (strong difference of importance)
7	Very strong or demonstrated importance of one factor with respect to another
9	Evidence of extreme difference of importance of one factor with respect to another

**Table 3:** Matrix of parameter of flood hazard for AHP pair wise comparisons.

Factors	Elevation	Drainage density	Slope	Rain fall	Land use	Soil
Elevation	1	1/3	3	1/3	5	3
Drainage density	3	1	3	1/3	5	5
Slope	1/3	1/3	1	1/3	5	3
Rain fall	3	3	3	1	7	5
Land use	1/5	1/5	1/5	1/7	1	1/3
Soil	1/3	1/5	1/3	1/5	3	1
Cumulative preferences in column	7.87	5.07	10.53	2.34	26.00	17.33

**Table 4:** Percentages of preferences and weight values' matrixes.

Factors	Elevation	DD	Slope	RF		LU		Soil		Weight (%)	
Percentage preference matrix											
Elevation	0.13	0.07	0.28	0.14		0.19		0.17		16.4	
DD	0.38	0.20	0.28	0.14		0.19		0.29		24.8	
Slope	0.04	0.07	0.09	0.14		0.19		0.17		11.8	
RF	0.38	0.59	0.28	0.43		0.27		0.29		37.4	
LU	0.03	0.04	0.02	0.06		0.04		0.02		3.4	
Soil	0.04	0.04	0.03	0.09		0.12		0.06		6.2	

	Elevation	DD	Slope	RF	LU		Soil		Sum		Ratio of sum over weight
Weight values matrix											
Elevation	0.16	0.08	0.36	0.12	0.17		0.19		1.70		6.86
DD	0.49	0.25	0.36	0.12	0.17		0.31		0.74		6.21
Slope	0.05	0.08	0.12	0.12	0.17		0.19		2.51		6.72
RF	0.49	0.74	0.36	0.37	0.24		0.31		0.21		6.34
LU	0.03	0.05	0.02	0.05	0.03		0.02		0.38		6.16
Soil	0.05	0.05	0.04	0.07	0.10		0.06		1.08		6.59

DD, RF, and LU represent the drainage density, rainfall, and land use, respectively.

**Table 5:** Weight of flood-generating factors after paired wise comparisons of AHP.

Flood-generating factors	Weight (%)
Rain fall	38
Drainage density	25
Elevation	16
Slope	12
Soil	6
Land use	3

**Table 6:** Flood hazard area coverages for different scenarios.

Scenario	Factor	Weight	Area flooded (ha)				
			Very low	Low	Moderate	High	Very high
			1	2	3	4	5
Scenario-2	Drainage density	20	3.7	965.16	5815.51	4446.94	131.80
	Elevation	20					
	Slope	20					
	Soil	20					
	Land use	20					

Scenario-3	Rain fall	20	0.01	244.32	4221.87	6756.94	140.05
	Elevation	20					
	Slope	20					
	Soil	20					
	Land use	20					

Scenario-4	Rain fall	20	0.30	490.32	8243.36	2609.40	19.76
	Drainage density	20					
	Slope	20					
	Soil	20					
	Land use	20					

Scenario-5	Rain fall	20	65.76	2545.66	7494.97	1237.02	19.76
	Drainage density	20					
	Elevation	20					
	Soil	20					
	Land use	20					

Scenario-6	Rain fall	20	0.02	413.31	6720.91	4380.36	22.64
	Drainage density	20					
	Elevation	20					
	Slope	20					
	Land use	20					

Scenario-7	Rain fall	20	0.01	1552.48	6801.32	2988.60	25.31
	Drainage density	20					
	Elevation	20					
	Slope	20					
	Soil	20					

## Data Availability

The data used to support the findings of this study are available upon reasonable request from the corresponding author.
